# Congenital peribronchial myofibroblastic tumor (CPMT): a case report with long term follow-up and next-generation sequencing (NGS)

**DOI:** 10.1186/s12887-023-04001-5

**Published:** 2023-04-20

**Authors:** Ping Zhou, Shuang Li, Weiya Wang, Yuan Tang, Lili Jiang

**Affiliations:** Department of Pathology, West China Hospital, Sichuan University, No. 37 Guo Xue Xiang, Sichuan Chengdu, China

**Keywords:** Congenital peribronchial myofibroblastic tumor, CPMT, Prenatal, Lung lesion, Next-generation sequencing

## Abstract

**Background:**

Congenital peribronchial myofibroblastic tumor (CPMT) is an extremely rare lung disease in infants. It shows benign behavior and has a favorable survival after surgical treatment. CPMT was reported only in cases. Here, we report the longest follow-up known case of CPMT and review the clinical, radiographic and histopathological features of the published literature.

**Case presentation:**

Ultrasound examination at 30 weeks of gestational age of a healthy 29-year-old female revealed a solid mass in the left lung. Computed tomography (CT) revealed a mass in the left lower lobe. The tumor was removed by lobectomy and pathologically diagnosed with CPMT. The tumor was composed of cartilage, spindle cells and oval cells. Vimentin was strongly positive. Smooth muscle actin (SMA) was positive in the spindle cells. The histopathologic and immunohistochemical features were similar to those in the literature. No *ETV6-NTRK3* fusion or *ALK* rearrangement was detected. Gene mutations in *JAK2* and *SMO* were detected by NGS. She is currently alive for 8 years with no evidence of disease recurrence.

**Conclusions:**

CPMT is a rare lung tumor in infants. Surgical treatment is recommended for CPMT. The prognosis after successful surgery is favorable. The final diagnosis was histopathologic findings. Due to its cellularity, mitotic activity and rapid growth, long-term follow-up should be strengthened. The present patient is alive and well for 8 years after the surgery without recurrence. Gene mutations in *JAK2* and *SMO* were detected, which may be associated with the formation of CPMT.

## Background

Lung lesions rarely occur in utero or infancy. With the development of ultrasonography, lung lesions in infants can be detected mostly by prenatal ultrasound examination. Congenital lung lesions included abnormalities in lung development, such as pulmonary sequestration (PS) and congenital pulmonary airway malformation (CPAM), and primary pulmonary tumors, such as pleuropulmonary blastoma (PB), congenital-infantile fibrosarcoma, and fetal lung interstitial tumor (FLIT).

Congenital peribronchial myofibroblastic tumor (CPMT) is an extremely rare lung disease in infants. The tumor was first reported in 1949 as “hamartoma” [1] and first used by the name “CPMT” by Mcginnis, M. in 1993 [2]. According to the 2021 World Health Organization (WHO) classification of thoracic tumors, CPMT is a solid fibroblastic/myofibroblastic tumor developing in utero or in infancy, composed of mitotically active but histological bland myofibroblasts arranged in fascicles. The etiology of CPMT is unknown and may develop during the early gestational age of pregnancy. The tumor is thought to originate from pluripotent mesenchymal cells around proximal bronchial branches [3]. Cytogenetically, only Alobeid, B et al. reported a complex rearrangement involving chromosomes 4, 8, and 10 in one case [4]. Clinically, CPMT is usually associated with respiratory distress [1, 4, 5], polyhydramnios [2, 6–11], fetal hydrops [2, 5, 7–9, 11–15] and intrauterine fetal demise [9, 16]. Lung lesions in infants can be detected by ultrasound examination and chest radiography, but the imaging features of CPMT are nonspecific, and the final diagnosis of CPMT is histopathologic findings.

CPMT was reported only in case reports, and there were just 24 cases reported in the published English-language literature [1–22]. Herein, we present the 25^th^ case and the second case in China and give a review of the literature with clinical, radiographic, and histopathologic characteristics.

## Case presentation

**Fig. 1:**
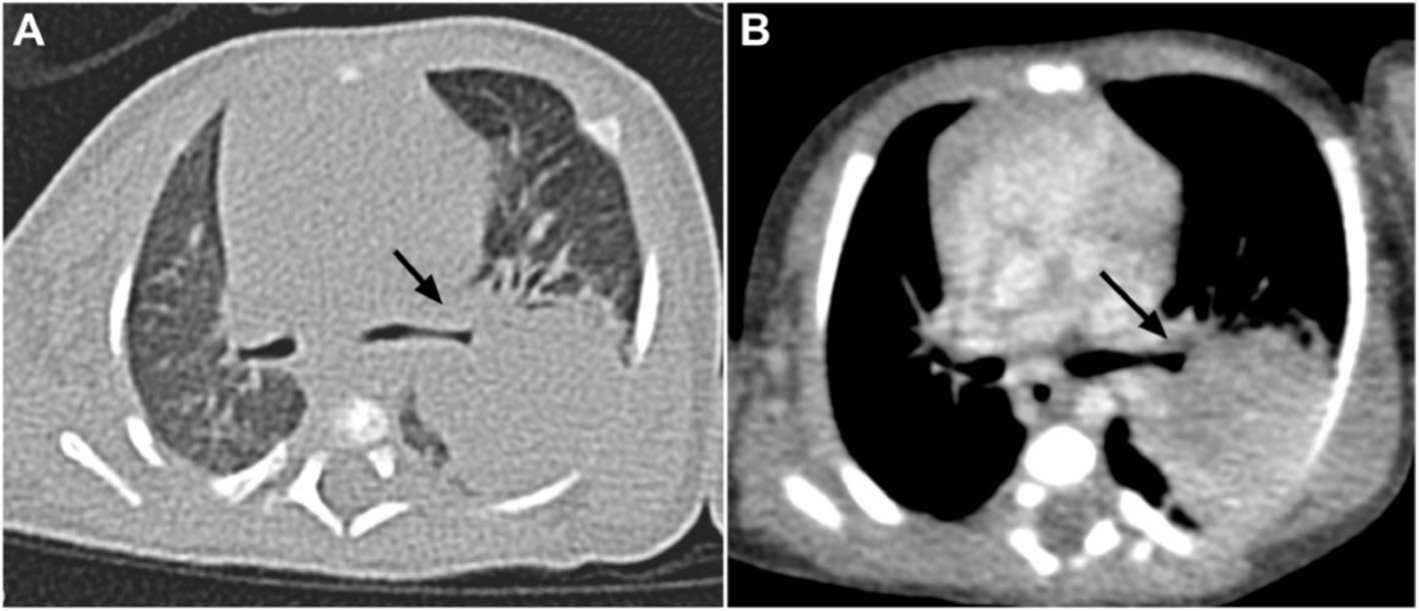
Computed tomography (CT) scan showed a solid lesion with irregular margin, which was closely related to bronchial airway in the left lower lobe (arrow indicates bronchi). The heart slightly shifted to the right thorax. (**A**, lung window; **B**, mediastinal window)

A healthy 29-year-old female, gravida two, para two (G2P2), her first child had died a few days after birth of respiratory distress syndrome (RDS). The present case was her second pregnancy. Prenatal ultrasonography was regularly scheduled, and she had no complicated pregnancy. She did not reveal polyhydramnios or hydrops fetalis. Ultrasound examination at 30 weeks of gestational age revealed a solid mass in the left lung, indicating CPAM. The neonate was delivered by cesarean section at the 34th week of gestation. The birth weight was 2.4 kg, and the newborn was 48 cm long. The Apgar scores were 10, 10, and 10 at 1, 5, and 10 min, respectively. She had no respiratory difficulty at birth. The infant was discharged home with stable vitals. The infant was suddenly cyanotic at 20 days after birth, and she was admitted to West China Hospital, Sichuan University. Chest computed tomography (CT) showed a large mass lesion in the left lower lobe, as shown in Fig**.** 1. The mass of the left lung was removed by lobectomy at the age of 22 days.

**Fig. 2:**
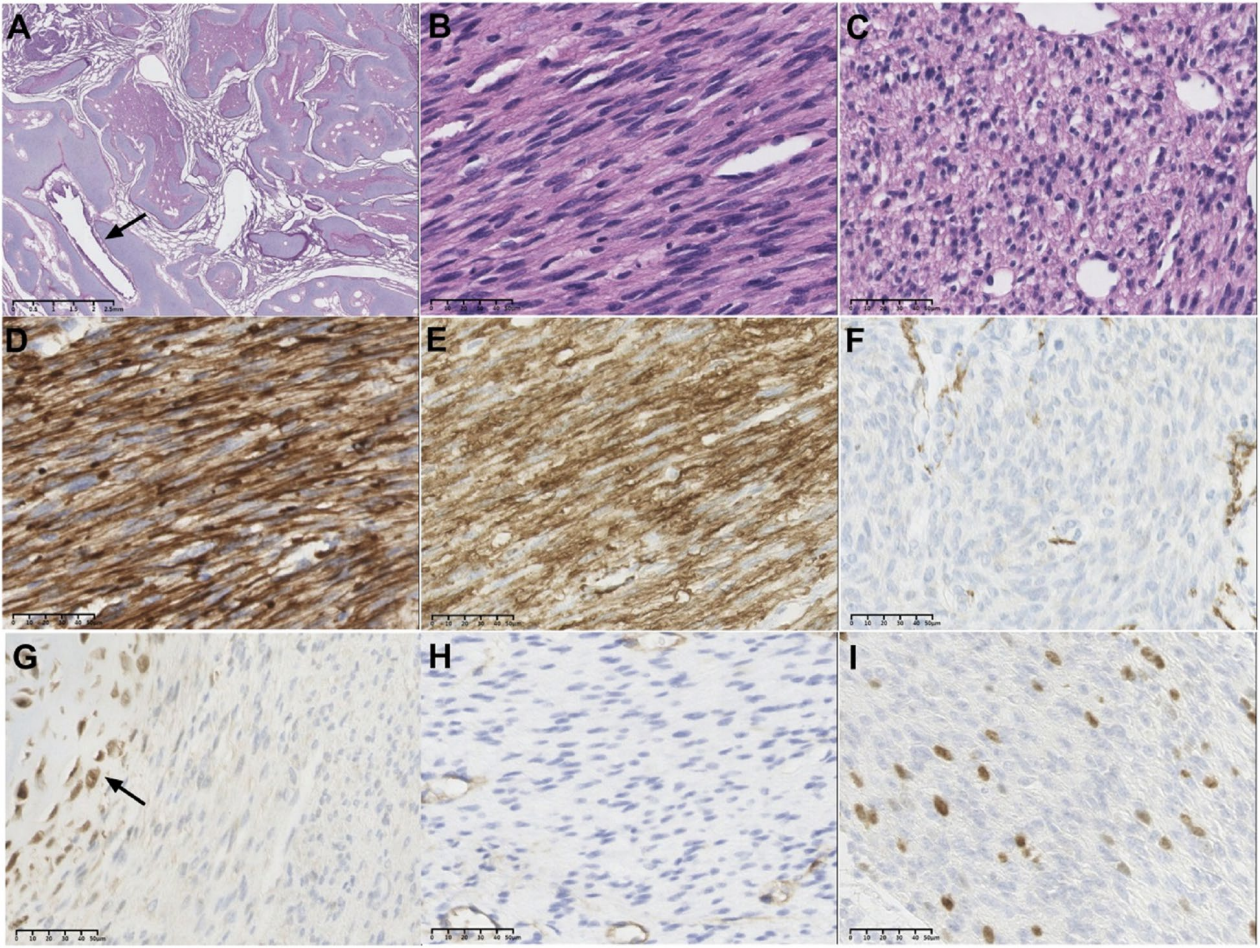
Microscopical images and immunohistochemical staining of CPMT. The tumor cells surrounded the bronchi with enlarged immature cartilage plates (**A**, arrow indicates bronchi). High-power magnification of spindle tumor cells (**B**) and oval tumor cells (**C**) (Hematoxylin and Eosin: magnification × 400). These tumor cells were diffusely positive for vimentin (**D**, magnification × 400). SMA was strongly positive in the spindle cells (**E**, magnification × 400), but oval cells showed faint to negative reactivity (**F**, magnification × 400). S100 was positive for cartilage and negative for spindle cells and oval cells (**G**, arrow indicates cartilage, magnification × 400). CD34 was negative for tumor cells (**H**, magnification × 400). The Ki67 index was approximately 8% (**F**, magnification × 400)

On gross examination, the lung showed a solid mass measuring 3.3 cm × 2.5 cm × 1.7 cm in size. Lung specimens were stained with hematoxylin and eosin for microscopic examination. Microscopically, the tumor surrounded the airway with peribronchial growth (Fig. 2A) and connected to the pleura. No capsule was visualized. The tumor was composed of immature cartilage, highly cellular spindle cells and oval cells interlacing fascicles (Fig. 2B and C). The cytoplasm of the spindle cells was eosinophilic. Nucleoli were inconspicuous. There was no central necrosis, anaplasia or pleomorphism. Mitotic figures were rare, and one per ten high-power fields was present in the case.

For immunohistochemical staining, strong vimentin positivity was observed in the spindle cells and oval cells (Fig. 2D). Smooth muscle actin (SMA) was strongly positive in the spindle cells, but oval cells showed faint to negative reactivity (Fig. 2E and F). S100 was positive for cartilage and negative for spindle and oval cells (Fig. 2G). CD34 was negative for tumor cells (Fig. 2H). The Ki67 index was approximately 8% (Fig. 2I). Pan-cytokeratin, epithelial membrane antibody (EMA), anaplastic lymphoma kinase 1 (ALK-1), desmin and OCT3/4 were negative.

**Fig. 3:**
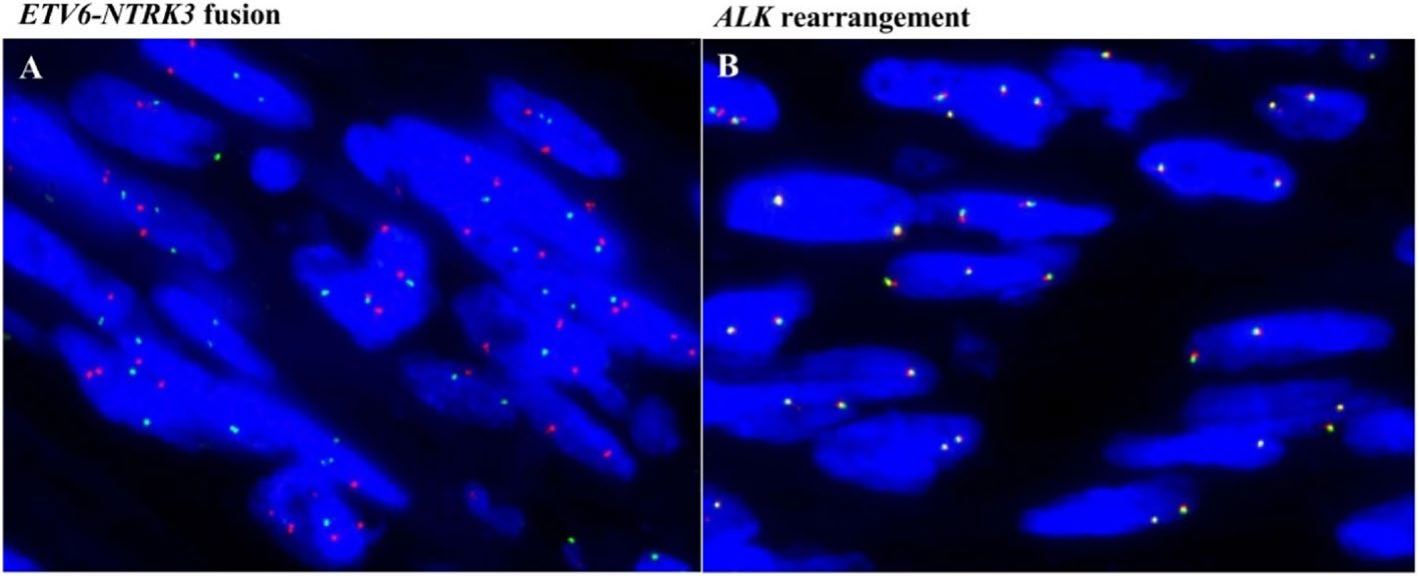
Fluorescence in situ hybridization (FISH) (magnification × 1000). No fusion of *ETV6-NTRK3* was detected (**A**). *ETV6-NTRK3* probes are a mixture of a spectrum green-labeled 12p13.2 probe (*ETV6*) and a spectrum red-labeled 15q25 probe (*NTRK3*). There was split green and red signal, without fused signal pair. *ALK* rearrangement was not detected (**B**). (< 15% of the tumor cells demonstrated split or single red signals)

No *ETV6-NTRK*3 fusion (Fig. 3A) or *ALK* rearrangement (Fig. 3B) was detected by fluorescence in situ hybridization (FISH) in the present case. Fifty-six next-generation sequencing (NGS) analyses were performed, and *JAK2* detected deletion mutations at sites ranging from 780 to 879 (c.780-879del), leading to a frameshift mutation in exon 7. *SMO* found a point mutation at the acceptor splicing site of exon 5 (c.1140 + 1G > A).

The final histological diagnosis was CPMT. No complications occurred in the postoperative period. The patient is now 8 years old, and she is doing well without further treatment after surgery. There was no clinical or radiologic evidence of recurrence or metastasis.

## Discussion and conclusions

Congenital peribronchial myofibroblastic tumor (CPMT) is classified as a neoplastic lesion, which was first described by Jones, C. J. in 1949 [1] as “Hamartoma of the Lung”. Since then, CPMT has been described as bronchopulmonary fibrosarcoma, leiomyosarcoma, hamartoma, and mesenchymal tumor in the literature [1, 5–8, 12, 17, 18]. In 1993, the term “congenital peribronchial myofibroblastic tumor, CPMT” was first suggested by McGinnis et al. [2]. CPMT is a solid fibroblastic/myofibroblastic tumor developing in utero or in infancy, composed of mitotically active but histological bland myofibroblasts arranged in fascicles.

The etiology of CPMT is unknown. The tumor is thought to originate from the pluripotent mesenchymal cells around proximal bronchial branches, which are destined to differentiate into cartilage and myofibroblasts to form the bronchial wall [3]. Cytogenetically, Alobeid, B et al. reported a complex rearrangement involving chromosomes 4, 8, and 10, and the karyotype of the tumor was 46,XX, t(8;10) (p11.2; p15), lns(10;4) (p15; q12iq21) or 46,XX, ins(8;4) (p11.2; q12q21), t(8;10) (p11.2; p15) [4]. *JAK2* mutation and *SMO* mutation were detected by 56-NGS in the present case, which may be associated with the formation of CPMT. Janus-activated kinase 2 (JAK2) is a member of the JAK protein family, and the activation of JAK2/STAT3 signaling is frequently detected in many tumors and plays an important role in oncogenesis, angiogenesis, and metastasis of many cancer diseases [23, 24]. Activating mutations in Smoothened (SMO) are tumorigenic and correlate with cell overgrowth [25]. Our results provide new insight into the formation of CPMT. This is the first known study to perform NGS. However, due to the long duration of tissue blocks, the quality of DNA was not satisfactory. Further sequecing of the CPMT is needed.

The histopathological features in the present case were similar to those in the published literature. The tumor surrounded the airway with peribronchial growth, composed of cartilage, spindle cells interlacing fascicles and oval cells. Strong positivity for vimentin was observed in the spindle cells and oval cell components, indicating that CPMT may originate from mesenchymal cells. Smooth muscle actin (SMA) positivity in the spindle cell component indicated that CMPT differentiated into smooth muscle tissue.

Lung lesions can be detected by ultrasonography, chest-ray or computed tomography, sometimes with mediastinal shift, but the imaging features of CPMT are nonspecific. It is characterized by rapid growth and is easily misdiagnosed as a malignant tumor. The final diagnosis of CPMT is histopathologic findings. The differential diagnosis included other congenital abnormalities and pulmonary tumors, such as pulmonary sequestration (PS), congenital pulmonary airway malformation (CPAM), chondromatous hamartoma, teratoma, pleuropulmonary blastoma (PB), fetal lung interstitial tumor (FLIT), inflammatory myofibroblastic tumor (IMT), congenital-infantile fibrosarcoma, monophasic fibrous type synovial sarcoma and so on. Most infantile fibrosarcomas have an *ETV6-NTRK3* fusion gene. Most pleuropulmonary blastomas include *DICER* mutations. Some inflammatory myofibroblastic tumors (IMTs) have *ALK* rearrangement fusion genes and positive immunoactivity for ALK-1. Some synovial sarcomas have the fusion gene *SS18*. In the present case, *ETV6-NTRK3* fusion and *ALK* rearrangement were not detected. Immunohistochemical staining of ALK-1 CD34, desmin and OCT3/4 was negative.

There were no symptoms in the present preterm case, with a lung mass approximately 3.3 in diameter. The present case received no further treatment after surgery. The patient is now 8 years old, and she is doing well, with no recurrence or metastasis. There were just 24 cases reported in the published English-language literature [1–22]. A summary of the cases is shown in Table 1. CPMT is a benign tumor, but its location, size and rapid growth can lead to clinical symptoms, such as respiratory distress [1, 4, 5], polyhydramnios [2, 6–11], fetal hydrops [2, 5, 7–9, 11–15] and intrauterine fetal demise [9, 16]. The ratio of males to females was 17:8. Most cases were preterm, indicating that CPMT mostly formed during the development of early pregnancy. The tumor size ranged from 3.3 cm to 12 cm. Except for three cases of elective termination and intrauterine fetal demise, there were seven infants without surgical treatment, and all seven cases died from birth to 17 days. Surgery is an effective treatment for CPMT. Lobectomy or pneumonectomy of the involved lung lobe is often recommended. Among the 15 cases that received surgical resection, the complications of surgery, such as massive bleeding, could be threatened for infants. There were two cases with intraoperative death [2, 20]. One case died 24 h after birth [6], during the surgical procedure, the pulmonary hilum was avulsed, and the infant exsanguinated. The other 12 cases had a favorable prognosis after successful surgical treatment, with no evidence of death ranging from 3 months to 8 years, and there was no report of recurrence or metastasis. CPMT has benign behavior, and chemotherapy or radiotherapy is not needed. The mitosis varied in cases. In addition, cellularity, mitotically active and growth rapidly were present in most cases, and long-term follow-up should be strengthened.

**Table 1:** The summary of the CPMT cases reported in the published English-language literature

Case	Years	Author	Polyhydramnios	Fetal hydrops	Gestational age at presentation	Sex	Tumor site	Tumor size(cm)	Diagnosis	Mitotic	Surgical treatment	Outcome
1	**1949**	**Jones, C. J** [1]	**None**	**None**	**28W**	**F**	**RUL**	**3.5**	**Hamartoma**	**NS**	**None**	**Died, 1 h**
2	**1958**	**Robb, D** [17]	**None**	**None**	**NS**	**M**	**LLL**	**6.5**	**Fibrosarcoma**	**Present but nor numerous**	**Thoracotomy**	**NED,10 months**
3	**1972**	**Guccion, J. G** [18]	**NS**	**NS**	**NS**	**M**	**LUL**	**6**	**Leiomyosarcoma**	**15/10HPF**	**None**	**Died at birth**
4	**1977**	**Haller, J. O** [5]	**None**	**Fetal hydrops**	**40**	**F**	**LLL**	**5.5**	**Congenital mesenchymal tumor**	**None**	**None**	**Died, 8 h**
5	**1985**	**Warren, J. S** [6]	**Polyhydramnios**	**None**	**30-33W**	**F**	**L lung**	**5.5**	**Congenital mesenchymal malformation**	**1–5/HPF**	**Pneumonectomy**	**Died, 24 h**
6	**1986**	**Jimenez, J. F** [7]	**Polyhydramnios**	**Fetal hydrops**	**36W**	**M**	**RLL**	**7.5**	**Leiomyosarcoma**	**Occasional**	**Lobectomy**	**NED, 34 months**
7	**1989**	**Pettinato, G** [12]	**None**	**Pleural effusion**	**NS**	**M**	**LUL**	**6**	**Primary bronchopulmonary fibrosarcoma**	**8–12/10HPF**	**Lobectomy**	**NED, 3 months**
8	**1989**	**Pettinato, G** [12]	**None**	**Pleural effusion**	**NS**	**M**	**RLL**	**7.5**	**Primary bronchopulmonary fibrosarcoma**	**8–12/10HPF**	**Lobectomy**	**NED, 6 years**
9	**1990**	**Khong, T. Y** [8]	**Polyhydramnios**	**Fetal hydrops**	**27W**	**M**	**RUL**	**4.5**	**Congenital mesenchymal malformation**	**Frequent**	**None**	**Died at birth**
10	**1993**	**Mcginnis, M** [2]	**Polyhydramnios**	**Fetal hydrops**	**33W**	**M**	**RUL**	**5**	**CPMT**	**1–5/10 HPF**	**Pneumonectomy**	**3 days (intraoperative death)**
11	**1997**	**Alobeid, B** [4]	**None**	**None**	**35W**	**F**	**RML, RLL**	**6**	**CPMT**	**0–3/10HPF**	**Thoracotomy**	**NED, 12 months**
12	**2005**	**Horikoshi, T** [16]	**None**	**Fetal hydrops**	**29W**	**M**	**LLL**	**6**	**CPMT**	**NS**	**None**	**Intrauterine fetal demise**
13	**2005**	**Reiss, A** [13]	**None**	**A small amount pleural effusion**	**25W**	**M**	**L lung**	**6.5**	**CPMT**	**0–4/HPF**	**None**	**Elective termination**
14	**2010**	**De Noronha, L** [9]	**Polyhydramnios**	**Fetal hydrops**	**24W**	**M**	**LLL**	**12**	**CPMT**	**Frequent but not abnormal**	**None**	**Intrauterine fetal demise**
15	**2011**	**Huppmann, A. R** [14]	**None**	**Fetal hydrops**	**23W**	**M**	**LLL**	**6.6**	**CPMT**	**Brisk**	**Lobectomy**	**NED, 6.5 years**
16	**2012**	**Acikalin, A** [10]	**Polyhydramnios**	**Hydrothorax**	**35W**	**M**	**RML**	**6.5**	**CPMT**	**4–5/HPF**	**Lobectomy**	**NED, 26 months**
17	**2013**	**Kim, Y** [19]	**None**	**None**	**4 postnatal weeks (42W)**	**F**	**RML, RLL**	**5.5**	**CPMT**	**8/10HPF**	**Right middle and lower bilobectomy**	**NED, 2 years**
18	**2014**	**Hotokebuchi, Y** [3]	**None**	**None**	**27W**	**M**	**L lung**	**NS**	**CPMT**	**10/10HPF**	**None**	**Died, 21 h**
19	**2014**	**Chang, C** [20]	**None**	**None**	**32.5W**	**F**	**LLL**	**5.2**	**CPMT**	**5/10HPF**	**None**	**Died, 17 days**
20	**2014**	**Chang, C** [20]	**None**	**None**	**Full term**	**F**	**RLL**	**6.5**	**CPMT**	**Rare mitoses**	**Surgical resection**	**Intraoperative death**
21	**2014**	**Calvo-Garcia, M. A** [11]	**Polyhydramnios**	**Fetal hydrops**	**29W**	**M**	**LUL**	**8.7**	**CPMT**	**NS**	**Lobectomy**	**NED, 17 months**
22	**2015**	**Brock, K** [21]	**None**	**None**	**8 postnatal weeks (46W)**	**M**	**RLL, RML**	**3.5**	**CPMT**	**Frequent**	**Pneumonectomy**	**NED, 16 months**
23	**2015**	**Tu, YA** [15]	**None**	**Fetal hydrops**	**27W**	**M**	**Mediastinum**	**6.9**	**CPMT**	**NS**	**None**	**Died, 2 days**
24	**2015**	**Xia, B** [22]	**None**	**None**	**28W**	**M**	**LLL**	**4.4**	**CPMT**	**NS**	**Lobectomy**	**NED, 12 months**
25	**2022**	**The current case**	**None**	**None**	**30W**	**F**	**LLL**	**3.3**	**CPMT**	**1/10HPF**	**Left lung bilobectomy**	**NED, 8 years**

*CPMT* Congenital peribronchial myofibroblastic tumor, *F* Female, *M* Male, *NS* Not specified, *L* Left, *R* Right, *RUL* Right upper lobe, *RLL* Right lower lobe, *RML* Right middle lobe, *LUL* Left upper lobe, *LLL* Left lower lobe, *NED* No evidence of disease, *W* Weeks, *HPF* High power field

CPMT is a rare lung disease, typically presenting as a solid lung mass in the fetal and neonatal period. CPMT is composed of immature cartilage and spindle cells in fascicles and oval cells. CPMT has a favorable survival after successful surgical resection. The present case of CPMT was detected at 30 weeks by ultrasound. The patient is doing well for 8 years after the surgery, with no recurrence. Gene mutations in *JAK2* and *SMO* were detected, which may be associated with the formation of CPMT.

## Data Availability

The datasets used and/or analyzed during the current study are available from the corresponding author on reasonable request.

## Abbreviations

CPMT: Congenital peribronchial myofibroblastic tumor
CT: Computed tomography
FISH: Fluorescence in situ hybridization
NGS: Next-generation sequencing
PS: Pulmonary sequestration
CPAM: Congenital pulmonary airway malformation
PB: Pleuropulmonary blastoma
FLIT: Fetal lung interstitial tumor
IMT: Inflammatory myofibroblastic tumor
